# Low Molecular Weight Hyaluronan Induces Lymphangiogenesis through LYVE-1-Mediated Signaling Pathways

**DOI:** 10.1371/journal.pone.0092857

**Published:** 2014-03-25

**Authors:** Man Wu, Yan Du, Yiwen Liu, Yiqing He, Cuixia Yang, Wenjuan Wang, Feng Gao

**Affiliations:** 1 Department of Molecular Biology, Shanghai Jiao Tong University Affiliated Sixth People’s Hospital, Shanghai, PR China; 2 Department of Molecular Biology and Clinical Laboratory, Shanghai Jiao Tong University Affiliated Sixth People’s Hospital, Shanghai, PR China; Ottawa Hospital Research Institute, Canada

## Abstract

Hyaluronan (HA), a large nonsulfated glycosaminogycan in the extracellular matrix, whose degraded fragments termed as low molecular weight hyaluronan (LMW-HA), has been reported as an important regulator of angiogenesis. However, little is known about the influence of LMW-HA on lymphangiogenesis. In this study, we try to explore the in vitro effects of LMW-HA on lymphangiogenesis and identify the underlying molecular mechanisms. Our results showed that LMW-HA stimulation significantly increased lymphatic endothelial cells (LECs) proliferation, migration and tube formation. Further experiments demonstrated that LMW-HA altered actin cytoskeleton rearrangement and increased the formation of intense stress fibers, lamellipodia and filopodia. Mechanistically, LMW-HA stimulation resulted in rapid tyrosine phosphorylation of protein kinase C α/βII (PKCα/βII) and extracellular-regulated kinase 1/2 (ERK1/2). Lymphalic vessel endotheilial hyaluronan receptor 1 (LYVE-1), a homologue of CD44, is the main cell surface receptor for HA in LECs. Blocking the binding interaction of LMW-HA with LYVE-1 using neutralizing anti-LYVE-1 antibodies significantly inhibited LECs proliferation, migration, tube formation and signal transduction induced by LMW-HA, suggesting that LMW-HA may play a critical role in the processes required for lymphangiogenesis through interactions with its receptor LYVE-1 and triggering intracellular signal cascades.

## Introduction

Lymphangiogenesis, the formation of lymphatic vessels, is a fundamental physiological process required for the development of the embryonic lymph system and regeneration of lymphatic vessels occuring in adult tissues during inflammation, wound healing, and tumor metastasis [1]. The basic process of lymphangiogenesis is composed of lymphatic endothelial cells (LECs) proliferation, migration and tube formation. Though considerable progress has been made during the past years, the molecular mechanisms regarding lymphangiogenesis are far less explored.

Hyaluronan (HA), an important and abundant component of the extracellular matrix, is a non-sulphated, negatively charged linear polymer of repeated disaccharide units of β (1, 4)-D glucuronic acid-β (1, 3) N-acetyl-D-glucosamine. Apart from its role in lubricating articulations and maintain the cohesion and structure of epithelium, HA has a crucial role in tumor progression. Most malignant solid tumors contain elevated levels of HA, and in some cases, HA levels were prognostic for malignant progression [2]. HA has been implicated in regulating tumor malignant behaviors, such as anchorage-independent growth [2], tumor cell motility [3], [4], and secretion of matrix metalloproteinase [5]. Moreover, many studies have proved that HA is a critical regulator of angiogenesis [6], [7]. Unfortunately, little is known about HA on its role in regulating lymphangiogenesis. A related study on HA treated tumors showed that HA promoted tumor lymphangiogenesis and intralymphatic tumor growth in vivo [8]. However, native HA or high molecular weight HA (HMW-HA) has no obvious effects on lymphangiogenesis in vitro [9].These conflicting results may be due to the biological characteristics of HA, in which the biological activities are largely depended on their molecular weight. Although the most widely distributed form of HA in normal tissues is HMW-HA with molecular weight varies from 10^5^to 10^7^Da, the low molecular weight HA (LMW-HA) can be synthesized *de novo* or generated by either hyaluronidase-mediated degradation or hydrolysis of native HA under pathological conditions [10]. HMW-HA plays a structural role and inhibits inflammation, immune response and angiogenesis, whereas LMW-HA or HA fragments exhibit pro-inflammatory effects and are proved to be potential stimulators to angiogenesis [11]–[15].

Lymphatic vessel endothelial hyaluronan receptor 1 (LYVE-1), having an overall homology of 43% with CD44, is a receptor for HA and expressed predominantly on LECs. HA appears to exert its biological effects through binding with specific cell-associated receptors. LMW-HA was proved to have the ability to interact with its receptors, such as CD44 or receptor for hyaluronan-mediated motility (RHAMM), substantially trigger series of intracellular signal transduction and promote angiogenesis [16]. The biological active LMW-HA is usually reported to be in molecular sizes between 3 and 10 disaccharides units that are not easy to digest further. Although CD44 and RHAMM are reported as the main receptors on vascular endothelial cells (VECs), they are mostly absent from lymphatic vessels, wherein the only known receptor for HA is LYVE-1[17], [18]. LYVE-1 is thus likely to play a major role in the regulation of HA on biological behaviors of LECs. Previous studies in breast cancer cell lines suggested that a high density of lymph vessels expressing LYVE-1 correlated with a high frequency of regional lymph node metastases [19], [20]. Nevertheless, whether LYVE-1 mediated HA-induced regulation of the biological behaviors within the lymphatic system is still unknown

In present study, we investigated the effects of defined length of LMW-HA mixtures (2-10 disaccharides) on binding with LYVE-1 in order to study the role of LMW-HA in lymphangiogenesis, including lymphatic endothelial cell morphology, proliferation, motility and tube formation. To clarify the underlying mechanisms of LMW-HA-induced LECs activiation, we next observed the influence of LMW-HA on the structure of actin filaments and the tyrosine phosphorylation of protein kinase C α/βII (PKCα/βII) and p42/44 extracellular signal regulated kinase (ERK1/2). Finally, neutralizing antibodies of LYVE-1 were used to verify the participation of LYVE-1 in LMW-HA-induced lymphangiogenesis.

## Results

### Expression of LYVE-1 on SVEC4-10

**Figure 1:**
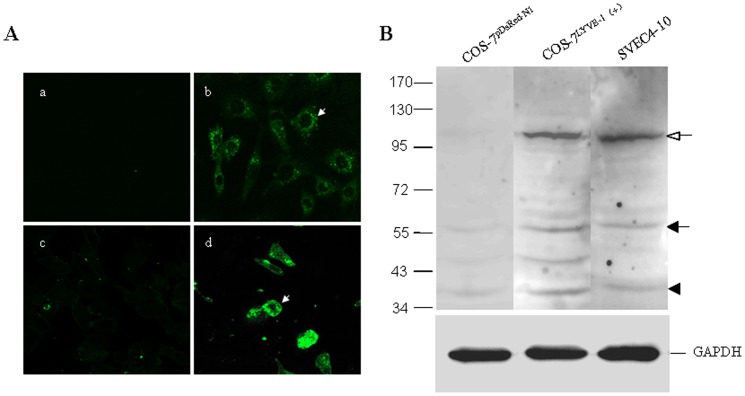
Expression of LYVE-1 on LECs. SVEC4-10, a SV40-immortalized endothelial cell line derived from mouse axillary lymph nodes, is a LEC-like cell line. (**A**)The expression of LYVE-1 on SVEC4-10 was assessed by immunofluorescence staining, confocal microscopic analysis and western bloting. Cells were grown on cover slips and fixed with 4% paraformaldehyde for 10 min. SVEC4-10 cells were stained with PBS (a) or anti-LYVE-1 mAb (b), followed by Alexa Fluxo 488-conjugated goat anti-rat IgG and visualized with a confocal microscope. COS-7^LYVE−1 (+)^ (d) and COS-7^pERFP−N1^ (c) were included as the positive control and negative control, respectively. The white arrows indicate the plasma membrane localization of LYVE-1. Magnification was ×600. (**B**) In addition, the cell lysates of SVEC4-10, COS-7^LYVE−1 (+)^ and COS-7^pERFP−N1^ were subjected to 7.5% SDS-PAGE followed by Western blot analysis using antibody to LYVE-1. The closed and opened arrows indicate the locations of the monomer and dimer of LYVE-1, respectively. The arrowhead indicates the location of a proteolytic product of LYVE-1. The slight difference of the location of LYVE-1 between COS-7^LYVE−1 (+)^ and SVEC4-10 cells may due to cell type-specific glycosylation [49]. Total GAPDH was used for normalization. Data were representative of three independent experiments.

SVEC4-10, a SV40-immortalized endothelial cell line derived from mouse axillary lymph nodes, has been reported as lymphatic endothelium [21]–[24]. Therefore, to more completely characterize the SVEC4-10 cell line, we assessed its expression of endothelial cell marker LYVE-1 by western bloting and immunofluorescent stain assay (**Figure 1** **and Figure S1**). Western blot analysis showed high expression of the lymphatic-specific marker LYVE-1 on SVEC4-10 and COS-7^LYVE−1 (+)^ but not on COS-7^pERFP−N1^ cells (**Figure 1B**). Immunofluorescent assay and confocal microscopy confirmed that LYVE-1 localizes to the plasma membrane and intracellular structures in SVEC4-10, which is consistent with previous studies (**Figure 1A**) [25]. SVEC4-10 cells also exhibit ultrastructural characteristic of LECs including sparse microvillous surface projections, overlapping intercellular junctions and abundant intermediate filaments [25]. They are LEC-like cells.

### Antibodies against LYVE-1 Inhibited LMW-HA-induced LECs Proliferation

**Figure 2:**
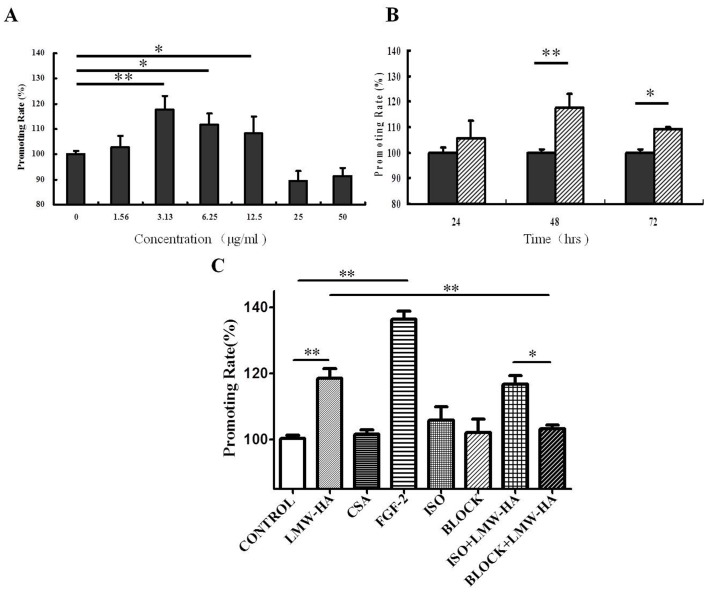
The effects of neutralizing antibodies to LYVE-1 on LMW-HA-induced proliferation of LECs. (**A**) Cells were incubated in a humidified atmosphere containing 5% CO_2_ at 37°C in the presence or absence of various concentrations of LMW-HA in 48h. (**B**) Cells were incubated with 3.13 μg/ml LMW-HA for different times. LMW-HA significantly enhanced the growth of LECs and the optimal concentration and time of LMW-HA were proved to be 3.13 μg/ml and 48h (**, *p*<0.01 compared with control group), which were chose for further study. (**C**) SVEC4-10 cells were pre-incubated with 10 μg/ml neutralizing antibody of LYVE-1 or isotype IgG for 12h, then LMW-HA was added to reach a final concentration of 3.13 μg/ml and incubated for another 48 h. The same volume of medium only instead of LMW-HA was added as blank control. Neutralizing anti-LYVE-1 antibodies or isotype IgG only were also included to eliminate the influence of antibodies themselves on the cells. Cells with FGF-2 (20 ng/ml, ***, *p*<0.001 compared with control group) or CSA (structural analogue of HA, 3.13 ug/ml, *p*>0.05) treatment for 48h was included as positive or negative control, respectively. Cell proliferation was evaluated by MTT assay and the promoting rate was calculated by comparing to the untreated control. The bars indicate means ± S.D. (n = 4). *, *p*<0.05. **, *p*<0.01. ***, *p*<0.001.

The effects of the LMW-HA on SVEC4-10 proliferation were analyzed by MTT assay. SVEC4-10 cells were treated with LMW-HA in various concentrations ranging from 1.56 to 50 μg/ml for different times. As shown in **Figure 2A**, LMW-HA significantly stimulated LECs proliferation in the concentration range of 3.13–12.5 μg/ml, which was the most at 3.13 μg/ml in 48h (118.5±5.84, *p*<0.01). In the time-dependent cell growth study, after treatment with 3.13 μg/ml LMW-HA the LECs proliferation was different at 24hrs and reached a peak at 48hrs (**Figure 2B**)**.**While LMW-HA had no significant effect on either COS-7 or NIH-3T3 cells in all the concentration examined (**Figure S2**). FGF-2, a well-described endothelium growth factor, was used as positive control in this experiment. The promoting rate of FGF-2 treated group (136.4±4.14) obviously increased compared to the blank control (100.0±2.31, *p*<0.001, **Figure 2C**). The negative control CSA had no significant effects on LECs proliferation (101.5±2.68, *p*>0.05, **Figure 2C**).

To determine if LYVE-1 participated in lymphangiogenesis induced by LMW-HA, SVEC4-10 cells were pre-incubated for 12h in the presence or absence of neutralizing monoclonal antibodies to LYVE-1 or isotype control IgG antibody (10 μg/ml), then LMW-HA were added to reach a final concentration of 3.13 μg/ml. 48 hours later, the proliferation rates of LECs were measured by MTT (**Figure 2C**). Compared with LMW-HA treated group, anti-LYVE-1 antibodies (103.2±2.26, *p*<0.05) but not isotype control (116.8±4.96, *p*>0.05) significantly inhibited LECs proliferation induced by LMW-HA. Significant difference was also found between ISO+LMW-HA and BLOCK+LMW-HA groups (*p*<0.05). Neutralizing anti-LYVE-1 antibodies and isotype IgG themselves had no significant effects on LECs proliferation (102.1±6.94 and 105.9±6.97, *p*>0.05).

### Antibodies against LYVE-1 Suppressed LMW-HA-promoted Cell Migration

**Figure 3:**
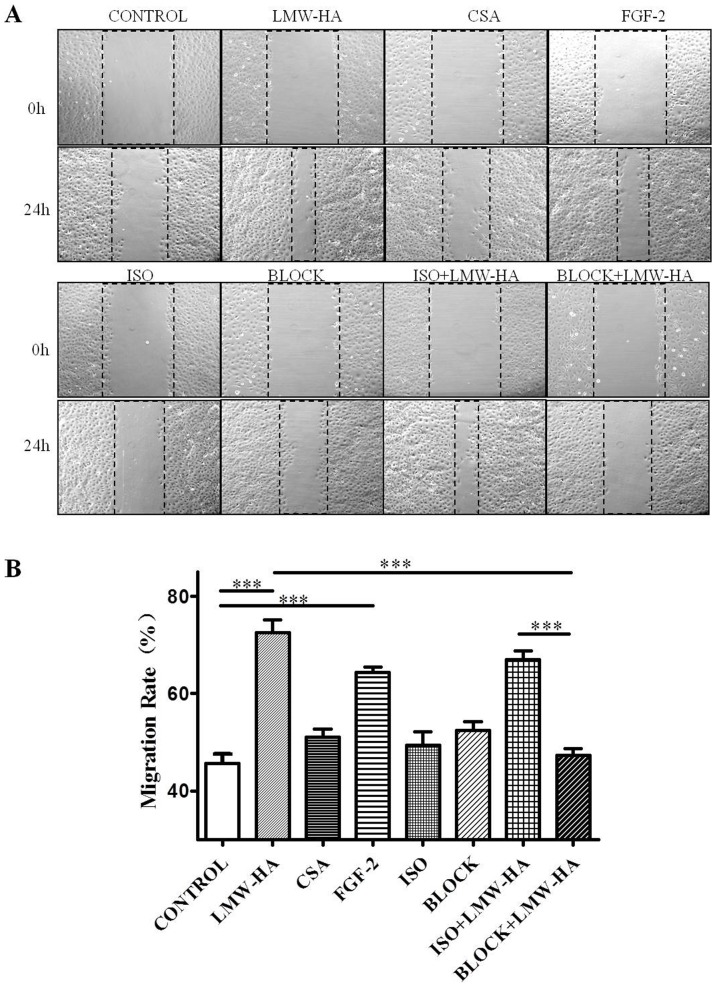
The effects of neutralizing antibodies to LYVE-1 on LMW-HA-induced migration of LECs. (**A**) SVEC4-10 cells were grown on cover slips to 100% confluent monolayers. Sterile pipette tips were used to scratch the confluent monolayer cells to form a 100 μm wound area, and then the cells were cultured for 24 h with or without 3.13 ug/ml LMW-HA. Wells with FGF-2 (20 ng/ml) or CSA (3.13 μg/ml) treatment was included as positive or negative control, respectively. To block LYVE-1/LMW-HA interaction, SVEC4-10 cells were pre-incubated with 10 μg/ml anti-LYVE-1 antibodies or isotype IgG_2A_ for 2h. Then LMW-HA was added to reach a final concentration of 3.13 μg/ml and incubated for another 24h. Neutralizing anti-LYVE-1 antibodies or isotype IgG only were also included to eliminate the influence of antibodies themselves on the cells. After incubation, the cells were fixed and observed by inverted microscope. All experiments were repeated at least three times and show a representative example. Magnification was ×100. (**B**) Migration rate (%) was analyzed by Image Pro Plus 6.0 software. ***, *p*<0.001 compared with the respective control group.

To determine the influence of LMW-HA on SVEC4-10 motility, wound healing assays were performed. The concentration of LMW-HA was chosen to be 3.13 μg/ml, as consistent with the optimal concentration in promoting SVEC4-10 proliferation. Cells were grown to 100% confluent monolayer on cover slips and were scratched to form a 100 μm wound with the sterile pipette tips. The cells were then incubated for 24 h with or without LMW-HA stimulation. SVEC4-10 cells at the leading edges of the wound migrated and spread to cover the wound (**Figure 3A**). Compared with blank control (45.71±4.29, both LMW-HA and FGF-2 significantly increased LECs migration (64.28±3.53 and 72.55±5.75, *p*<0.001, **Figure 3B**). In contrast, there was no significant difference between CSA and the blank control group (50.98±5.09, *p*>0.05) and LMW-HA exerted no significant effect on the migration of either COS-7 or NIH-3T3 cells (**Figure S3**).

Neutralizing anti-LYVE-1 antibodies significantly decreased LECs migration promoted by LMW-HA (47.25±3.97, *p*<0.05). While isotype control IgG exert no inhibitory effects on LMW-HA induced LECs migration (66.95±3.75, *p*>0.05). Moreover, cell migration rate in BLOCK+LMW-HA group was significantly lower than ISO+LMW-HA group (*p*<0.001). Neutralizing anti-LYVE-1 antibodies and isotype IgG themselves had no significant effects on LECs migration (52.41.±3.56 and 49.37±6.36, *p*>0.05).

### Antibodies against LYVE-1 Decreased LMW-HA-enhanced Tube Formation

**Figure 4:**
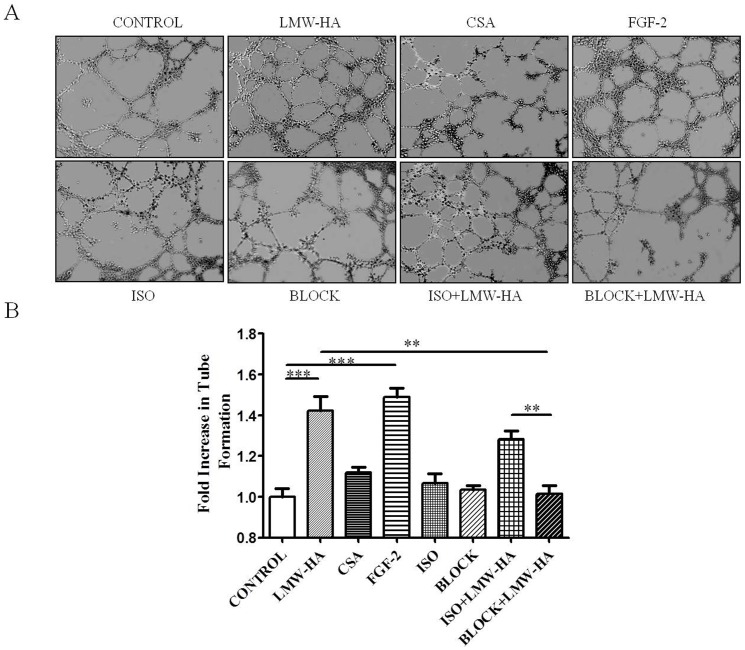
The effects of neutralizing antibodies to LYVE-1 on LMW-HA-induced LECs tube formation. (**A**) SVEC4-10 cells were plated on matrigel in 96-well plates in complete medium contain LMW-HA (3.13 μg/ml), FGF-2 (20 ng/ml), CSA (3.13 μg/ml) or medium only. In some cases, cells were pre-treated with neutralizing anti-LYVE-1 antibodies or isotype IgG for 30min and then LMW-HA was added to reach a final concentration of 3.13 μg/ml and incubated for another 24 h. (**B**) Tube formation was quantitated by determining the total length of tubes. All experiments were repeated at least three times and show a representative example. Data points are fold increase over control. Magnification was×100. **, *p*<0.01. ***, *p*<0.001.

The differentiation and organization of EC into vascular tubes are critical steps in the process of angiogenesis and lymphangiogenesis. Using a model of endothelial tube formation on Matrigel, we found that after incubation overnight in a basal medium only, SVEC4-10 cells formed few tubular structures (**Figure 4A**). In contrast, in the presence of 3.13 μg/ml LMW-HA, the tubular network was formed more significantly (1.42-fold, *p*<0.05) compared to the blank control. Cells treated with FGF-2 also showed increased formation of capillary-like networks (1.49-fold, p *p*<0.05), while CSA had no significant effects on LECs tube formation (**Figure 4B**).

To investigate the role of LYVE-1 in LMW-HA-enhanced tube formation, neutralizing anti-LYVE-1 antibodies were used. No decrease of LMW-HA-induce tube formation was observed with isotype control IgG-treated group but there was a significant decrease after anti-LYVE-1 antibodies treatment compared to LMW-HA alone. Significant difference in tube formation was also observed between ISO+LMW-HA and BLOCK+LMW-HA groups (*p*<0.01). Neutralizing anti-LYVE-1 antibodies and isotype IgG themselves had no significant effects on LECs tube formation.

### LMW-HA Remodeled the Structure of Actin Filaments

Actin filaments mediate signal transduction in cells and undergo dynamic reorganization or remodeling in response to stimulation. Several activated forms of kinases remodel the structure of actin filaments to form the cell motile structures and change the structures of actin stress fibers. Here we sought to determine whether stimulation by LMW-HA has the capability to reorganize the structure of actin filaments in LECs.

**Figure 5:**
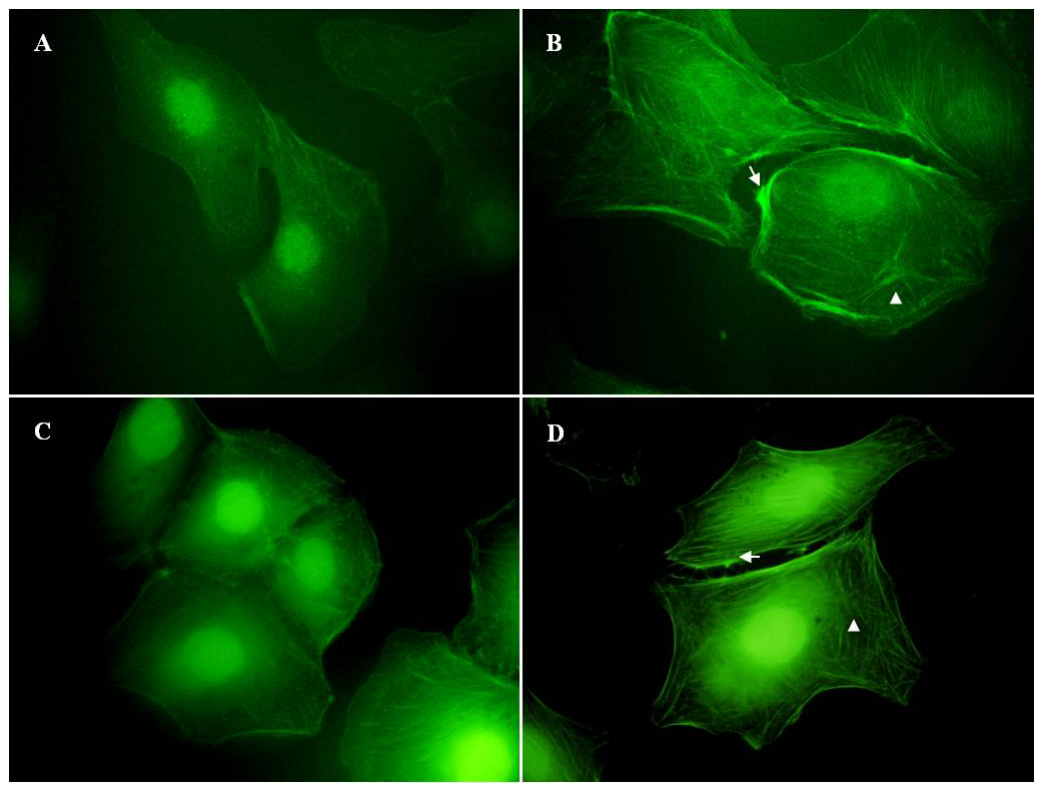
LMW-HA stimulation induces the reorganization of actin filaments in LECs. SVEC4-10 cells were grown on cover slips and serum-starved overnight. Then the cells were incubated with (**B**) LMW-HA (3.13 μg/ml) or (**A**) medium only for 24h. Cells treated with (**D**) FGF-2 (20 ng/ml) or (**C**) CSA (3.13 μg/ml) was used as positive or negative control, respectively. After stimulation, cells were fixed on cover slips and stained with FITC-phalloidin. Fluorescence microscope was used to analyze the rearrangement of actin filaments and the changes in cell morphology. All experiments were repeated at least three times and show a representative example. Magnification was ×100. The arrows indicate stress fibers. The arrowheads indicate rosette-like dots.

It was found that the actin stress fibers were well organized in the unstimulated SVEC4–10 cells, for example, cells were evenly spread out, and few cell motile structures and rosette-like dots were found at the cell leading edges and within cell bodies (**Figure 5A**). Upon stimulation with LMW-HA (**Figure 5B**) or FGF-2 (**Figure 5D**) for 24 hours, more intense F-actin stress fibers arranged in spike-like protrusions resembling microvilli-like structures or dissociated to form the rosette-like structures around the cell body and protruded the cell membrane at the leading edging to form cell motile structures such as lamellipodia and filopodia. No obvious change of actin filaments was found after CSA treatment (**Figure 5C**).

### Antibody against LYVE-1 Inhibited LMW-HA-intensified ERK and PKC Phosphorylation

**Figure 6:**
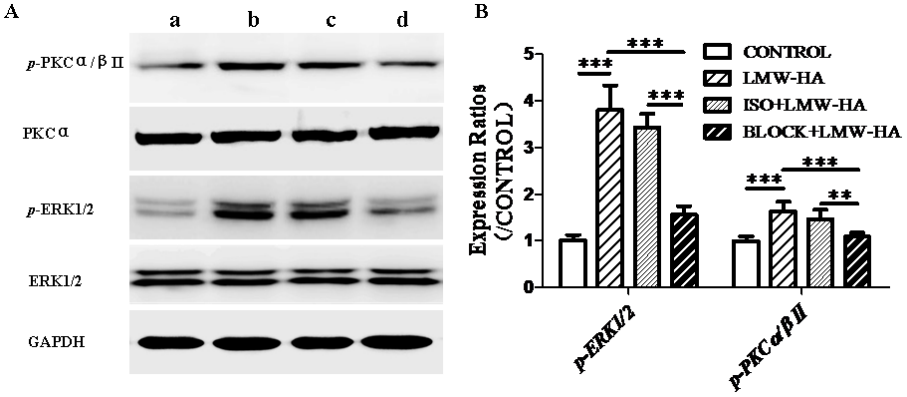
The effects of neutralizing antibodies to LYVE-1 on LMW-HA-induced PKC/MAPK activation. (**A**) Phosphorylated versions and total of PKC and ERK were analyzed by Western blotting. SVEC4-10 cells were incubated with LMW-HA (**b**, 3.13 μg/ml) or serum free medium only (**a**) for 20min. In some cases, cells were pre-treated with neutralizing anti-LYVE-1 antibodies (**d**) or isotype IgG (**c**) for 12h and stimulated with LMW-HA (3.13 μg/ml) for another 20min. After stimulation, cells were then analyzed by Western blotting. Total PKCα and ERK1/2 in addition to GAPDH were used for normalization. Data are representative of three independent experiments. (**B**) Expression ratios were analyzed by Image Pro Plus 6.0 software. **, *p*<0.01. ***, *p*<0.001.

As previously reported, LMW-HA can stimulate ECs proliferation and wound healing by inducing intracellular signal transduction, including activation of PKC and ERK1/2, followed by early response gene activation [26]. PKC can activate MAP kinase through PKC-MAP kinase pathway [27]. To further understand the intracellular mechanism through which LMW-HA enhances LECs proliferation and migration, we next studied the influence of LMW-HA on activation of PKC and ERK1/2 signaling, which have been regarded as crucial regulators of EC proliferation and migration [28]. Western blot analysis revealed that compared to control cells (1.00±0.10), addition of LMW-HA increased the expression of the phosphorylated forms of PKCα/βII (1.63±0.21, *p*<0.001, **Figure 6**). We also found that phospho-ERK1/2 was increased fourfold (4.06±0.22, *p*<0.001, **Figure 6**) after LMW-HA treatment compared to untreated cells.

To identify the role of LYVE-1 in LMW-HA-stimulated signal transduction, we investigated the effects of neutralizing anti-LYVE-1 antibodies on ERK and PKC phosphorylation triggered by LMW-HA. Compared to LMW-HA treated group, antibodies against LYVE-1 significantly decreased PKC and ERK phosphorylation (1.09±0.09 and 1.56±0.19, *p*<0.001), while isotype control IgG exerted no inhibitory effects (1.46±0.21 and 3.36±0.25, *p*>0.05).

## Discussion

Lymphangiogenesis is actively involved in a number of physiological and pathological processes, including wound healing and tissue inflammation [1]. More recently, lymphangiogenesis has also been implicated in lymphatic metastasis of various human cancers, such as carcinomas of the breast, colon, and prostate as well as melanoma [29]–[31]. Studies of tumor models in animals and clinicopathological data have indicated that lymphangiogenesis in the vicinity of solid tumors may contribute to lymphatic metastasis [30], [31].

HA is a ubiquitous and major component of the extracellular matrix, which accumulates in sites of cell division and rapid matrix remodeling occurring during embryonic morphogenesis, inflammation and tumorigenesis. Some reports showed that malignant tumor cells often exhibit elevated levels of not only HA itself but also hyaluronidase, which renders tumor cells to synthesis HA with different molecular weight [32]. HA and hyaluronidase are closely related to tumor metastasis. Specifically, HMW-HA was shown to provide an attachment and hydration pathway enabling cellular nutrients to enter the primary tumor and exert an inhibitory effect on angiogenesis. Subsequently, the presence of LMW-HA that appears to be primarily generated by highly invasive cancer cells turns the angiogenic switch to commence neovascularization and long-term maintenance of the tumor mass [33]. On the other hand, clinical studies have demonstrated that the presence of LMW-HA may be associated with the bone-metastasizing ability of bone metastasizing renal tumors of childhood (BMRTC) [34]. LMW-HA level in the sera of BMRTC was significantly increased, while was barely detectable in the sera of patients with no bone metastasis. Following surgery in BMRTC patients, both serum HA and LMW-HA levels fall to a value within normal ranges [34]. It is likely that LMW-HA is associated with malignant phenotype, angiogenesis and tumor metastasis. Until now, however, little is known about the role of LMW-HA in lymphangiogenesis occurred in tumor growth and metastasis. In this research, we studied the effects of LMW-HA on a mouse lymphatic endothelial cell line, SVEC4-10, which is generally regarded as an ideal in vitro model for lymphangiogenesis study [24], [25], and described for the first time that LMW-HA may be an important factor in regulating lymphangiogenesis.

Repertoires of cell processes, including LEC proliferation, migration, differentiation and tube formation, are essential for lymphangiogenesis. Studies have demonstrated LMW-HA may interact with various cells and initiate a program of gene expression leading to cell proliferation and migration [18], [35].In agreement with previous studies, our experiment showed that activation of LECs was induced by LMW-HA, including a significant increase in LECs proliferation, migration and tube formation. The results suggested that LMW-HA may actively induce lymphangiogenesis through promoting LECs proliferation, migration and tube formation (**Figure 2** **,** **3** **and** **4**). We also found that low concentrations of LMW-HA promoted LECs proliferation, but excessive concentrations (>50 μg/ml) caused a decrease of proliferation (**Figure 2A**), which were similar with its effects on angiogenesis in the previous reports [36].

Dynamic regulation of the filamentous actin (F-actin) cytoskeleton is critical to numerous physical cellular processes, including cell adhesion, migration and division. Stress fibers and associated focal adhesions in cells constitute a contractile apparatus that regulates cell motility and specific cytoskeletal rearrangements of cells have been implicated in cell migration and adhesion [37], [38]. In this report, we demonstrate that LMW-HA stimulation could induce the remodeling of F-actin in LECs, increase intense F-actin stress fibers, and induce the formation of cell motile structures (**Figure 5B**). As a main receptor for HA on VECs, CD44 was reported to interact with cytoskeletal proteins resulting in cytoskeleton rearrangement in response to HA binding. CD44 is mostly absent from lymphatic vessels, in which the predominant receptor for HA is LYVE-1 [19], [39]. However, whether LYVE-1 has the similar function in interaction with cytoskeleton causing downstream remodeling is still unknown [40]. Our data suggested that LMW-HA may stimulate cytoskeletal rearrangements, resulting in promotion of LECs motility and triggering intracellular signal transduction associated with mitogenesis.

To further understand the intracellular mechanism through which LMW-HA enhances LECs proliferation, migration and tube formation, we determined the effect of LMW-HA on tyrosine phosphorylation of two important members of Mitogen-activated protein kinase (MAPK) families, including PKC and ERK1/2. MAPK families play an important role in complex cellular programs like proliferation, differentiation, development, transformation, and apoptosis. The classical ERK family (p42/44 MAPK), an intracellular checkpoint for cellular mitogenesis, plays a pivotal role in the control of cell cycle progression. Previous studies have shown that LMW-HA stimulated second messenger activity in BAEC involved PKC and ERK1/2, accelerated G1/S transition and resulted in mitogenesis [28]. We found that LMW-HA also significantly enhanced ERK1/2 phosphorylation in LECs (**Figure 6**). In addition, an increased phosphorylation of PKCα/βII was observed (**Figure 6**), which is known to be important for dissociation of spectrin from the actin cytoskeleton and is critical in determination of cell shape and movement [41]. Previous reports have shown that activation of PKCα, β1, β2 and ε were important in the formation of *p*-ERK1/2 and cell mitogenesis [28]. Activation of ERK1/2 and mitogenesis occurred mainly through PKCα, whereas PKCβ1/2 had a much weaker effect but was necessary for efficient wound recovery in LMW-HA-treated cells [28]. Our study suggested that the activation of PKC/ERK signals is likely to be involved in the enhanced cell proliferation and cell motility induced by LMW-HA.

LYVE-1, 43% structurally homologous to CD44, is largely restricted to LECs [42]. In the past few years, LYVE-1 is widely used as one of the most specific lymphatic markers. Previous studies have demonstrated that LYVE-1 is not merely a useful tracing marker for the lymphatics but has specific biological functions including cell-surface retention of growth factors in the PDGF superfamily [25], regulation of lymphatic intercellular junctions [43], and mediation of the adhesion of tumor cells to lymphatic vessels [44]. Although the function of LYVE-1 attracts much attention, its role in lymphangiogenesis and lymphatic metastasis has not been extensively studied. The biological effects of HA depend on binding interactions with its specific receptors [18].CD44 and RHAMM are the receptors for HA on VECs, and both of them can mediate the signaling required for these processes relevant to angiogenesis. However, the main receptor for HA on LECs is LYVE-1. Thus LYVE-1 is a potential candidate for mediating LMW-HA-induced regulation of biological behaviors in LECs. In this study, we try to determine such role of LYVE-1 in lymphangiogenesis. The data showed that LMW-HA-enhanced LECs proliferation, migration and tube formation could be blocked by neutralizing anti-LYVE-1 antibodies. In addition, neutralizing anti-LYVE-1 antibodies also abrogated the intracellular signals triggered by LMW-HA, including phosphorylation of PKCα/βII and ERK1/2. The results proved that LMW-HA-induced lymphangiogenesis was partly dependent on its binding interaction with LYVE-1.

In conclusion, our study suggested that LMW-HA may promote the formation of lymphangiogenesis by enhancing LECs proliferation, migration, tube formation and cytoskeletal rearrangement. Most importantly, we revealed the possible role of LYVE-1 in the mediation of lymphangiogenesis induced by LMW-HA. We have also indentified novel components of the downstream signaling pathway associated with lymphangiogenesis induced by LMW-HA. In particular, LMW-HA significantly promoted phosphorylation of PKCα/βII and ERK1/2. Further studies need to be undertaken to fully understand the intracellular mechanisms responsible for LMW-HA induced lymphangiogenesis, which may be useful in the identification of potential targets for modulation of lymphangiogenesis in a variety of diseases associated with abnormal LEC growth. In addition, since no experimental procedures have been explored with regard to primary lymphatic endothelial cells in our studies, a well-established primary LEC model is expected for further confirmation. Further work in this area is ongoing in our laboratory.

## Materials and Methods

### Reagents

Native HMW-HA, chondroitine sulfate A (CSA) and FITC-phalloidin were obtained from Sigma-Aldrich (St. Louis, MO, USA). LMW-HA was prepared as described previously [45], [46], which was a mixed fraction of average molecular weight of 2.5×10^3^ composed of 3 to 10 disaccharide units that were fractionated from testicular hyaluronidase type 1-S (Sigma, St. Louis, MO, USA ) digests of hyaluronan sodium salt (Sigma, St. Louis, MO, USA). Fibroblast growth factor-2 (FGF-2) was purchased from PeproTech Inc. (Rocky Hill, NJ, USA). Matrigel was from BD Biosciences (Bedford, MA, USA). Monoclonal or polyclonal antibodies against total ERK1/2, phospho-ERK1/2 (Thr202, Thr204), phospho-PKCα/βII (Thr638, Thr641) and GAPDH were obtained from Cell Signal (Beverly, MA, USA). Monoclonal antibodies against PKCα were from Epitomics (Burlingame, CA). Alexa Fluxo 488-conjugated goat anti-rat IgG, HRP-conjugated rabbit anti-mouse and HRP-conjugated goat anti-rabbit IgG were all from Jackson ImmunoResearch Laboratories Inc. (West Grove, Pennsylvania,USA). Mouse neutralizing monoclonal antibody to LYVE-1 and rat IgG_2A_ isotype control were from R&D systems (Minneapolis, MN, USA). Pierce ECL western bloting substrate was from Thermo scinentific (Rockford, IL, USA). All other chemicals were of reagent grade or higher.

### Cell Culture

The C3H/HeJ immortal mouse endothelial cell line SVEC4-10 (CRL-2181), isolated from the axillary lymph nodes, was purchased from the American Type Culture Collection. SVEC4-10 cells were cultured in Dulbecco’s modified Eagle’s medium (DMEM, Gibco, Invitrogen Corporation, Carlsbad, CA, USA) supplemented with 10% fetal bovine serum in a humid atmosphere with 5% CO_2_ at 37°C. Cell lines were maintained in culture flasks until they reached confluence, at which time they were treated for 2–4 min at 37°C with 0.25% trypsin and 0.02%EDTA and were passaged to new flasks at a dilution of 1:6. After reaching 80% of confluence, cells were collected for the following experiments.

### Characterization of SVEC4-10 Cell Line

To indentify the expression and distribution of LYVE-1 on SVEC4-10 cells, an indirect immunofluorescent stain assay was performed. SVEC4-10 cells were grown on cover slides. Cells were washed briefly with phosphate-buffered saline (PBS), fixated in 4% paraformaldehyde for 10 min, then permeabilized using Triton X-100 (0.1%v/v in PBS) for 10min at room temperature. The cells were incubated with anti-LYVE-1 antibodies overnight at 4°C, and then stained with Alexa Fluxo 488-conjugated goat anti-rat IgG for 1h in the dark at room temperature. The expression of LYVE-1 was observed under confocal microscope (Nikon A1, Tokyo, Japan). Cells stained with PBS instead of the first antibody were used as blank control. COS-7^LYVE−1 (+)^ cells, in which *LYVE-1* gene was cloned and co-expressed with the red fluorescent protein (RFP) in COS-7 cells, was included as the positive control. COS-7^pDsRed−N1^ cells that transfected with a control empty pDsRed-N1 vector was used as the negative control.

SVEC4-10 cells were harvested and homogenized in ice-cold sodium dodecylsulfate (SDS) lysis buffer. Total cell lysates were collected and equal quantities of protein were separated by 7.5% SDS-PAGE and blotted onto a PVDF membrane. The PVDF membranes were blocked with Tris-buffered saline (TBS) containing 5% skimmed milk powder for 1 h, incubated with anti-LYVE-1 mAb (R&D) at 4°C overnight. Then the membranes were washed with 1×Tris-buffered saline/Tween-20 (TBS/T) buffer for three times (5 min each time) and incubated with HRP-conjugated polycolonal secondary antibody for 1h at room temperature. The membranes were developed with the enhanced plus chemiluminescence assay (Pierce, USA), according to manufacturer’s instructions. COS-7^LYVE−1 (+)^ and COS-7^pERFP−N1^ were included as the positive control and negative control, respectively.

### Cell Proliferation Assay

The effects of LMW-HA on SVEC4-10 proliferation were measured using 3-[4, 5-dimethylthiazol-2-yl]-2, 5-dipheniltetrazolium bromide (MTT) assay. Briefly, the collected cells were suspended in DMEM medium with 10% fetal bovine serum, and then seeded at a density of 3×10^3^ cells/well in the 96-well plate. Then, cells were incubated in the presence or absence of various concentrations of LMW-HA (1.56, 3.13, 6.25, 12.5, 25 and 50 μg/ml) in a fully humidified atmosphere containing 5% CO_2_ at 37°C for 24h, 48 h and 72h. The optimal concentration and time of LMW-HA were proved to be 3.13 μg/ml and 48h, which were chose for further study. COS-7 and NIH-3T3 cells with parallel treatment were also included to confirm the general effect of LMW-HA on LECs.

To evaluate the role of LYVE-1 in LMW-HA stimulated proliferation, SVEC4-10 cells were pretreated with or without anti-LYVE-1 antibodies (10 μg/ml) for 12h. Then cells incubated as described above with LMW-HA (3.13 μg/ml) for 48 h in the presence or the absence of anti-LYVE-1 antibodies (10 μg/ml). Isotype-matched nonimmune IgG was added to parallel cultures to assess the specificity of the anti-LYVE-1 antibodies. After incubation, 20 μl of MTT regent was added to each well. The absorbance was then measured at 570 nm by a Microplate Reader (Bio-Rad, Model 550). Cells treated with neutralizing anti-LYVE-1 antibodies or isotype-matched nonimmune IgG only were added to eliminate the influence of antibodies themselves on the cells. Wells with FGF-2 (20 ng/ml) or CSA (structural analogue of HA, 3.13 ug/ml) treatment were included as positive or negative control, respectively. Six individual wells were conducted for each group in three independent experiments. Cell proliferation rate (percentage of control)  =  (A570 nm of the treatment group/A570 nm of the control) × 100%.

### Cell Migration Determination

The wound healing assays were performed according to the methods described by Meng et al. [47]. SVEC4-10 cells were grown on cover slips in 6-well plates to 100% confluent monolayers and then scratched to form a 100 μm “wound” using sterile pipette tips. Medium was replaced with DMEM containing 1% fetal calf serum and cells were then incubated for a further 24h with or without LMW-HA (3.13 μg/ml), in triplicate. COS-7 and NIH-3T3 cells with parallel treatment were also included to confirm the general effect of LMW-HA on LECs.

To clarify whether LYVE-1 mediated LMW-HA-induced cell migration, SVEC4-10 cells were pretreated with or without anti-LYVE-1 antibodies (10 μg/ml) for 2h. Then cells incubated as described above with LMW-HA (3.13 μg/ml) for 24 h in the presence or the absence of anti-LYVE-1 antibodies (10 μg/ml). Isotype-matched nonimmune IgG was added to parallel cultures to assess the specificity of the anti-LYVE-1 antibodies. Then cells were fixed on cover slips with formalin. Movement of cells into the denuded area was quantified using an Image-Pro plus version 5.0 computerized image analysis systems (Media Cybernetics, Georgia). Cells treated with neutralizing anti-LYVE-1 antibodies or Isotype-matched nonimmune IgG only were added as control. Wells with FGF-2 (20 ng/ml) or CSA (3.13 μg/ml) treatment were included as positive or negative control, respectively. For each slide, 18 fields of view were examined at random. The lesion area in each field of view was measured and using the data from time 0 (T0), the wound area (Tt, 24h post-injury) was then converted to give mean migration rate (%) from 3 identically treated slides. The equation is migration rate (%)  =  [1– (wound area at Tt/wound area at T0)] ×100%. The experiments were repeated at least three times.

### Tube Formation Assay

Eighty microliters Matrigel (BD Biosciences) were polymerized in the wells of a 96-well plate at 37°C for 30 min. Serum-starved (8 h) SVEC4-10 cells (4×10^4^) were dispensed into each well in 100 μl DMEM medium with 10% fetal bovine serum, with or without LMW-HA (3.13 μg/ml).

To explore the role of LYVE-1 in LMW-HA stimulated tube formation, cells were pretreated with or without anti-LYVE-1 antibodies (10 μg/ml) for 30min. Then cells incubated as described above with LMW-HA (3.13 μg/ml) for 16 h in the presence or the absence of anti-LYVE-1 antibodies (10 μg/ml). Isotype-matched nonimmune IgG was added to parallel cultures to assess the specificity of the anti-LYVE-1 antibodies. After 16 h, cord morphogenesis of LECs was assessed by phase-contrast microscopy. Cells treated with neutralizing anti-LYVE-1 antibodies or Isotype-matched nonimmune IgG only were added as control. Wells with FGF-2 (20 ng/ml) or CSA (structural analogue of HA, 3.13 μg/ml) treatment were included as positive or negative control, respectively. Tube-formation data were quantified by measuring the total length of tube structures in a given field of observation using the Image Pro Plus 6.0 software (Media Cybernetics, Houston, TX, USA), then averaged from a triplicate set of samples for each experimental condition.

### Cell Morphology Observation

To demonstrate the change of actin filaments, after treatment with or without LMW-HA (3.13 μg/ml), cells were fixed in 4% paraformaldehyde for 10 min, then permeabilized using Triton X-100 (0.1%v/v in PBS) for 10min at room temperature [48], followed by labeling with FITC-phalloidin for 20min and mounting to the slides with Fluoromount (Fisher). Wells with FGF-2 (20 ng/ml) or CSA (3.13 μg/ml) treatment was included as positive or negative control, respectively. Images were observed under a fluorescent microscope.

### Western Blotting Assay

SVEC4-10 cells (80% confluence) were cultured in 6-well plates in complete medium. After attachment, the medium was replaced with serum free medium containing LMW-HA (3.13 μg/ml) or medium only for 20min. To determine whether LYVE-1 was involved in LMW-HA-stimulated signal transduction, cells were pretreated with or without anti-LYVE-1 antibodies (10 μg/ml) for 12h, and then stimulated by LMW-HA (3.13 μg/ml) for 20min in the presence or the absence of anti-LYVE-1 antibodies (10 μg/ml). Isotype-matched nonimmune IgG was added to parallel cultures to assess the specificity of the anti-LYVE-1 antibodies. Cells were then harvested and homogenized in ice-cold sodium dodecylsulfate (SDS) lysis buffer. Total cell lysates were collected and equal quantities of protein were separated by 12% SDS-PAGE and blotted onto a PVDF membrane. The PVDF membranes were blocked with Tris-buffered saline (TBS) containing 5% skimmed milk powder for 1 h, and then incubated at 4°C overnight with PKCα mAb, phospho-PKCα/βII (Thr638/641) pAb, ERK1/2 pAb or phospho-ERK1/2 mAb, respectively. After that, the membranes were washed with 1×Tris-buffered saline/Tween-20 (TBS/T) buffer for three times (5 min each time) and incubated with HRP-conjugated polycolonal secondary antibody for 1h at room temperature. The membranes were developed with the enhanced plus chemiluminescence assay (Pierce, USA) according to manufacturer’s instructions. Images were analyzed by Image pro-Plus6.0 software. The phosphorylated versions (*p*-PKCα/βII, *p*-ERK1/2) were normalized to their respective total protein (PKCα, ERK1/2) in addition to the GAPDH loading control.

### Statistical Analysis

The data were presented as Mean±SD. All statistical analysis was performed using SPSS11.0. Comparison among different groups (n≥3) used analysis of variance (ANOVA). The Student-Newman-Keuls (SNK) test was used for the comparison between two different groups. *P* less than 0.05 were considered significant.

## Supporting Information

Figure S1 **Expression of LYVE-1 on COS-7 cells.** COS-7 cells were transiently transfected with either full-length LYVE-1 cDNA in the expression vector pDsRed-N1 (COS-7^LYVE−1 (+)^; **G**, **H** and **I**) or with a control empty pDsRed-N1 vector (COS-7^pDsRed−N1^; **D**, **E** and **F**). Untransfected COS-7 cells (**A**, **B** and **C**) were used as a control. The transfection was analyzed by surface immunofluorescent staining with rat polyclonal LYVE-1 antibody and Alexa Fluxo 488-conjugated goat anti-rat IgG.
(JPG)

Figure S2 **The effects of LMW-HA on cell proliferation of COS-7 and NIH-3T3.** **(A)** COS-7 or **(C)** NIH-3T3 cells were incubated in a humidified atmosphere containing 5% CO_2_ at 37°C in the presence or absence of various concentrations of LMW-HA in 48h. **(B)** COS-7 or **(D)** NIH-3T3 cells were incubated with 3.13 μg/ml LMW-HA for different times. Cell proliferation was evaluated by MTT assay and the promoting rate was calculated by comparing to the untreated control. Data are representative of three independent experiments. The bars indicate Mean ± S.D. (n = 3).
(JPG)

Figure S3 **The effects of LMW-HA on cell migration of COS-7 and NIH-3T3.** **(A)** COS-7 or **(B)** NIH-3T3 cells were grown on cover slips to 100% confluent monolayers. Sterile pipette tips were used to scratch the confluent monolayer cells to form a 100 μm wound area, and then the cells were cultured for 24 h with or without 3.13 ug/ml LMW-HA. After incubation, the cells were fixed and analyzed by inverted microscope. Magnification was ×100. All experiments were repeated at least three times and show a representative example. **(C)** Migration rate (%)  =  [1- (wound area at Tt/wound area at T0)] ×100%. The bars indicate means ± S.D.
(JPG)
