# Supplementary figures and images for: Low Molecular Weight Hyaluronan Induces Lymphangiogenesis through LYVE-1-Mediated Signaling Pathways
Source: PLoS One. 2014 Mar 25;9(3):e92857. doi: 10.1371/journal.pone.0092857 (PMC3965470; doi:10.1371/journal.pone.0092857)

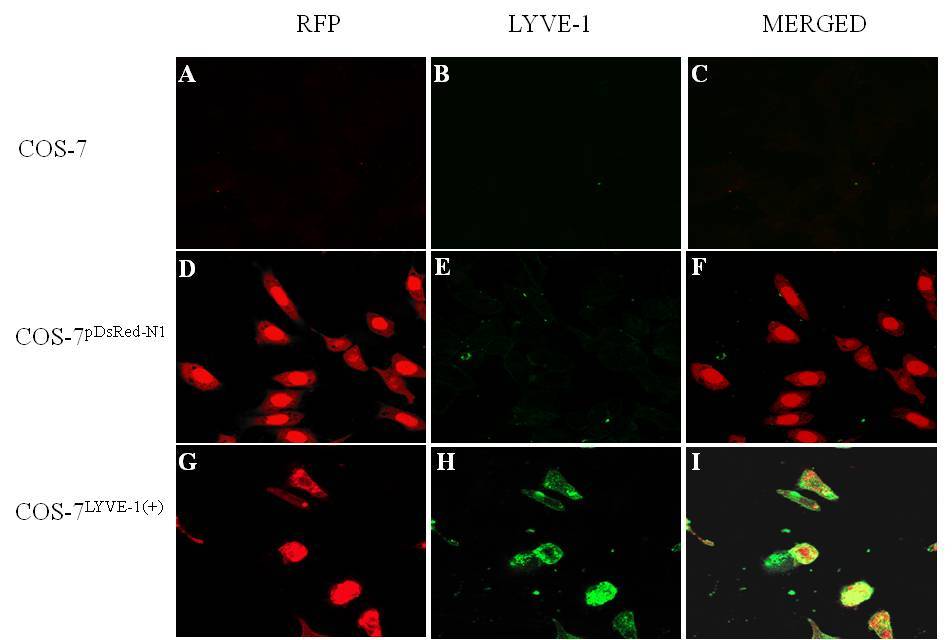

Supplement: Figure S1 — Expression of LYVE-1 on COS-7 cells. COS-7 cells were transiently transfected with either full-length LYVE-1 cDNA in the expression vector pDsRed-N1 (COS-7LYVE−1 (+); G, H and I) or with a control empty pDsRed-N1 vector (COS-7pDsRed−N1; D, E and F). Untransfected COS-7 cells (A, B and C) were used as a control. The transfection was analyzed by surface immunofluorescent staining with rat polyclonal LYVE-1 antibody and Alexa Fluxo 488-conjugated goat anti-rat IgG. (JPG) [file pone.0092857.s001.jpg]

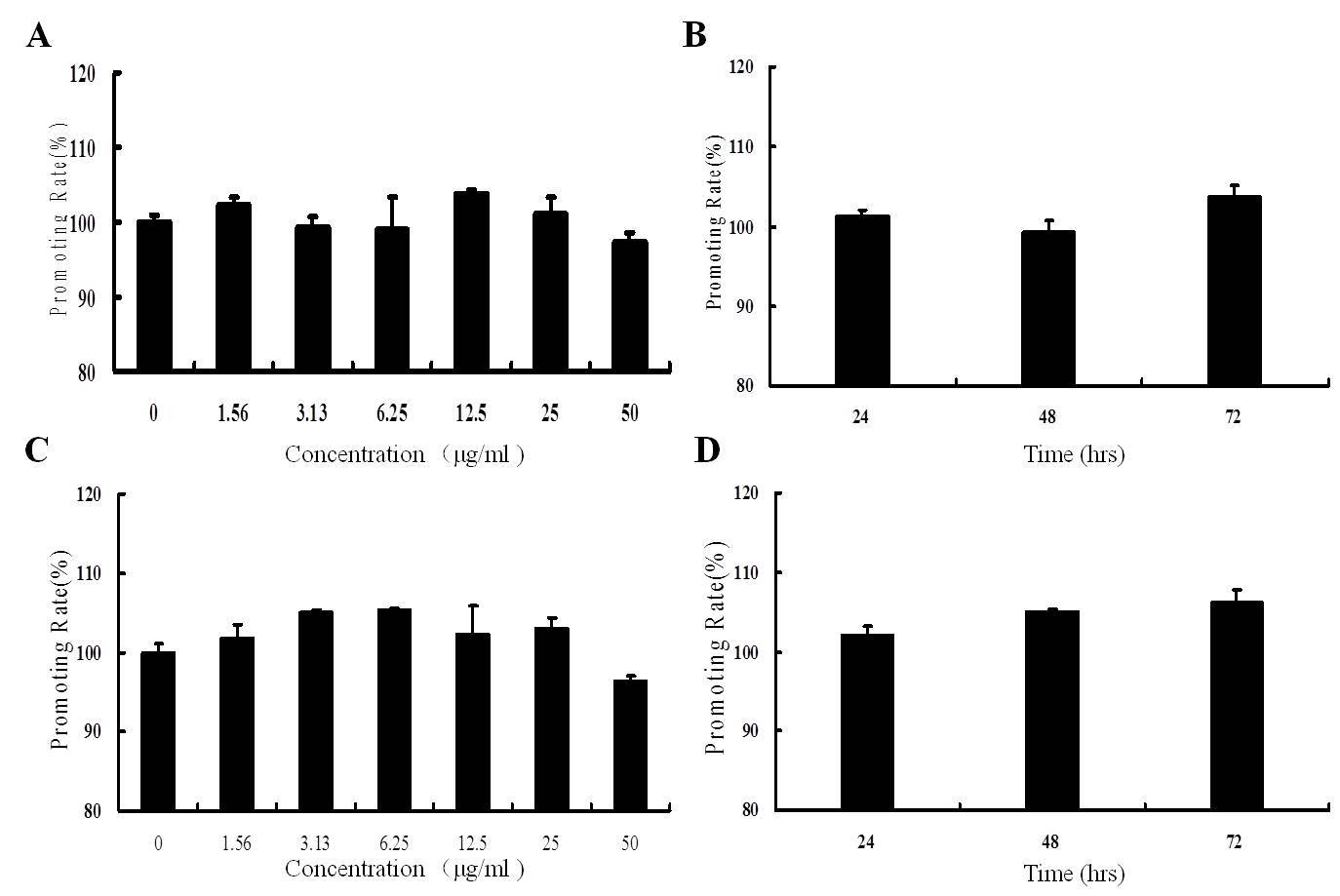

Supplement: Figure S2 — The effects of LMW-HA on cell proliferation of COS-7 and NIH-3T3. (A) COS-7 or (C) NIH-3T3 cells were incubated in a humidified atmosphere containing 5% CO2 at 37°C in the presence or absence of various concentrations of LMW-HA in 48h. (B) COS-7 or (D) NIH-3T3 cells were incubated with 3.13 μg/ml LMW-HA for different times. Cell proliferation was evaluated by MTT assay and the promoting rate was calculated by comparing to the untreated control. Data are representative of three independent experiments. The bars indicate Mean ± S.D. (n = 3). (JPG) [file pone.0092857.s002.jpg]

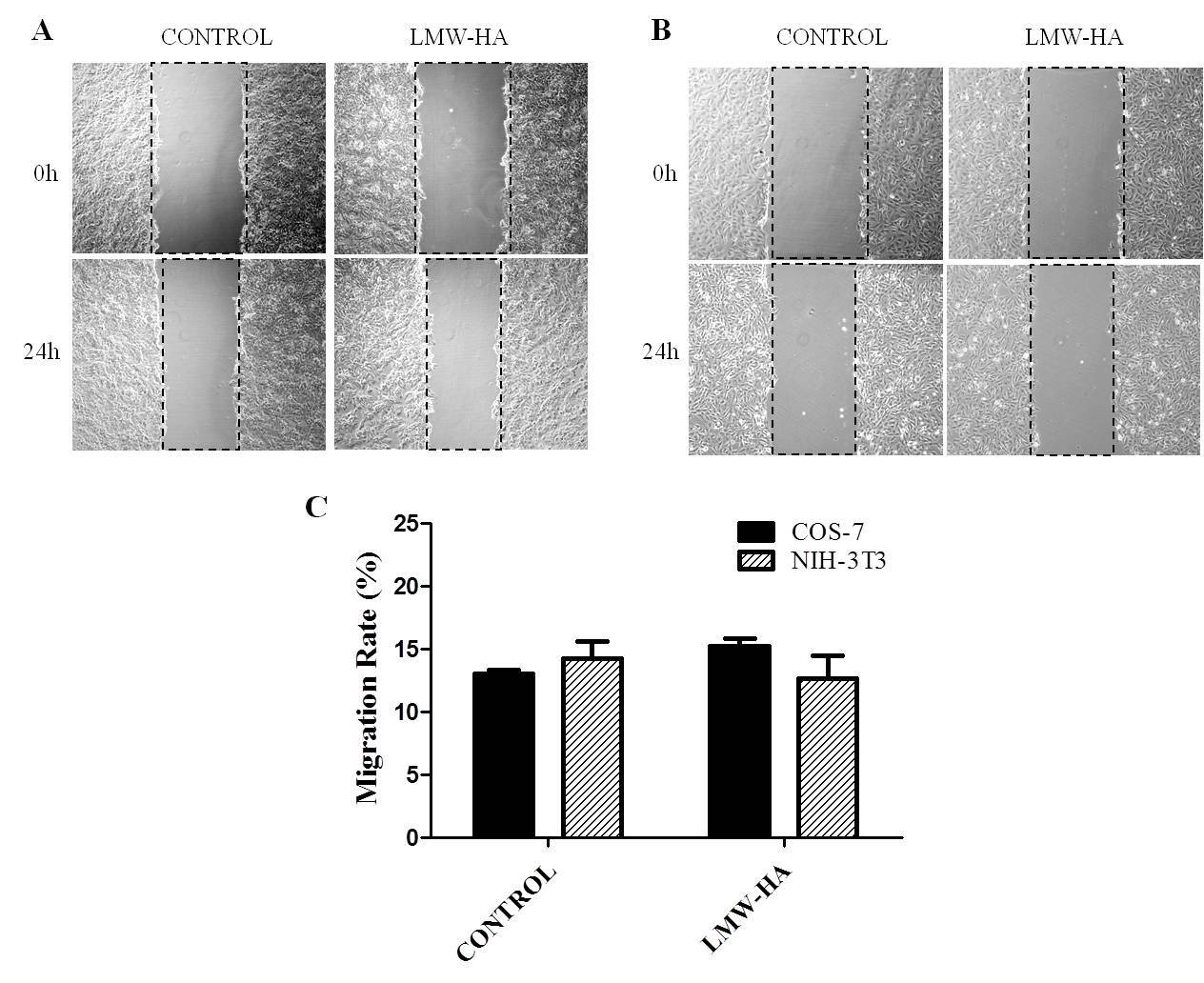

Supplement: Figure S3 — The effects of LMW-HA on cell migration of COS-7 and NIH-3T3. (A) COS-7 or (B) NIH-3T3 cells were grown on cover slips to 100% confluent monolayers. Sterile pipette tips were used to scratch the confluent monolayer cells to form a 100 μm wound area, and then the cells were cultured for 24 h with or without 3.13 ug/ml LMW-HA. After incubation, the cells were fixed and analyzed by inverted microscope. Magnification was ×100. All experiments were repeated at least three times and show a representative example. (C) Migration rate (%) = [1- (wound area at Tt/wound area at T0)] ×100%. The bars indicate means ± S.D. (JPG) [file pone.0092857.s003.jpg]
